# Gut Microbiota Signatures and Potential Mediators in the Trajectory of Age-related Macular Degeneration: A Phased Atlas by Genetic Inference

**DOI:** 10.7150/ijms.119158

**Published:** 2026-02-04

**Authors:** Yifan Zhou, Zhenyu Wang, Chen Huang, Xiaotong Yu, Junfu Chen, Xinhao Jiang, Jialong Dong, Qing Peng, Long Li, Xudong Song, Xinmin Lu

**Affiliations:** 1 Department of Ophthalmology, Shanghai Tenth People's Hospital, School of Medicine, Tongji University, 301 Middle Yanchang Road, Shanghai, China; 2 Department of Ophthalmology, Shanghai Sixth People's Hospital, School of Medicine, Shanghai Jiao Tong University, Shanghai, China; 3 Beijing Tongren Eye Center, Beijing Tongren Hospital, Beijing Ophthalmology and Visual Science Key Lab, Capital Medical University, NO.1 Dongjiaominxiang Street, Dongcheng District, Beijing, China, 100730; 4 Center of Basic Medical Research, Institute of Medical Innovation and Research, Peking University Third Hospital, No. 49 North Garden Road, Haidian District, Beijing, China; 5 Department of Functional Intestinal Diseases, General Surgery of Shanghai Tenth People's Hospital, Tongji University School of Medicine, 301 Middle Yanchang Road, Shanghai, China; 6 School of Clinical Medicine, Shanghai University of Medicine & Health Sciences, Shanghai 201318, China

**Keywords:** age-related macular degeneration, gut microbiota, Mendelian randomization, mediation analysis

## Abstract

**Purpose:**

To depict an atlas of stage-stratified gut microbiota (GM) signatures and intermediatory metabolites, inflammatory proteins, and immune cell traits, governing the AMD trajectory.

**Methods:**

We deployed bidirectional two-sample Mendelian randomization (TSMR) integrating GWAS data of 207 GM taxa from the Dutch Microbiome Project (N = 7,738), and multiple AMD stages/subtypes, including 'Macular degeneration (senile) of retina', 'Early AMD', 'Disease progression to GA/CNV', 'Dry AMD includes GA', and 'Wet AMD', encapsulating the disease trajectory (N > 410,000), complemented by multivariable MR (MVMR) mediation analysis of 1,400 circulating metabolites, 731 immune cell traits, and 91 inflammatory proteins.

**Results:**

We identified 12/8/5/2/9/8 genetically predicted causal GM taxa of various AMD stages/subtypes as a stage-stratified GM signature across the AMD trajectory, among which *g.Ruminococcaceae* and *s.Ruminococcaceae_bacterium_D16* were the sole shared GM taxa in triple AMD stages, while *s.Bacteroides eggerthii*, *c.Gammaproteobacteria*, *s.Dorea* and *s.Ruminococcus_obeum* influence dual AMD stages. Bidirectional analysis revealed that *f.Streptococcaceae*, *g.Erysipelotrichaceae_noname*, *g.Streptococcus*, *s.Streptococcus_thermophilus*, *g.Ruminococcaceae_noname*, and *s.Ruminococcaceae_bacterium_D16* exhibited genetically reciprocal causation with AMD. We also proposed that *Firmicutes* may exhibit stage-specific duality depending on their constituent members and AMD stages. Several understudied GM from *p.Actinobacteria* and *p.Verrucomicrobia* have been implicated as AMD-associated taxa for the first time. Key metabolites, immune cell traits, and inflammatory proteins were established as significant mediators of GM-AMD links.

**Conclusions:**

This first phased atlas uncovers GM effects over the AMD course, identifying potential microbial and biochemical targets for intervening in disease development.

## 1. Introduction

Age-related macular degeneration (AMD), the leading cause of irreversible central vision loss in elderly populations 1, retains unresolved pathophysiological complexity despite decades of research 2. Emerging insights into the "eye-gut axis" implicate gut microbiota (GM) dysbiosis as a potential AMD modulator 3, 4, with proposed mechanisms involving immune-metabolic cross-talk 5, 6. Intriguingly, GM interactions align with AMD risk factors spanning aging, metabolic disorders, and lifestyle behaviors 7. Yet, critical knowledge gaps persist in this burgeoning area: a) Methodological constraints: The vast majority of existing studies are confined to 16sRNA GM sequencing reports with limited sample sizes, restricting taxonomic resolution to genus level and lacking the statistical power to detect clinically meaningful associations. More importantly, clinical observations merely document microbial abundance alterations without characterizing pathogenic/protective functional roles of specific taxa; b) Temporal blindness: Cross-sectional sampling of patients in single AMD stage fails to capture dynamic GM shifts across AMD trajectory 8, which is a limitation common to clinical observation of GM in other diseases as well; c) Confounding entanglement: Inherent susceptibility of GM to AMD risk factors (diet, medications, comorbidities) creates insurmountable confounding in observational studies, perpetuating causal ambiguity. Additionally, clinical trials concerning GM-AMD may encounter cost and ethical challenges. As such, ascertaining causal disease-associated GM taxa and investigating GM's impacts throughout the disease course are desiderata in this field, before further mechanistic research and therapeutic development take out 7.

Building upon the current literature, the present study seeks to bridge these knowledge gaps by an integrated analytical framework of Mendelian Randomization (MR), a robust analytical technique for establishing causality through genetic instrumental variables, widely accepted as an alternative method for assessing the causal effects of related factors on diseases 9. While AMD possesses a multifactorial etiology, the genetic predisposition to AMD remains highly non-negligible, as evidenced by familial and twin studies 10-13. Key AMD risk loci such as CFH (chromosome 1q32), ARMS2/HTRA1 (10q26), and APOE (19q13) have been identified through genetic susceptibility research 14, 15. Notably, gene-environment synergism, such as the smoking-LOC387715 interactions, may elevate AMD risk, underscoring the genetic interplay between susceptibility genes and environmental risk factors 16-18.

The present study adopts an intricate design to utilize the SNP-heritability in AMD and GM. First, we obtained GM data from the Dutch Microbiome Project (DMP), the largest GWAS dataset that provides GM information from phylum to species level. Next, to investigate the GM role in a lengthy disease trajectory, we employed a diverse array of GWAS datasets of various AMD stages/subtypes, spanning preclinical senile degeneration of macula, early AMD, SNPs representing disease progression to two major advanced events: choroidal neovascularization (CNV)/geographic atrophy (GA), and the advanced wet/dry subtypes, to represent the whole disease course. We employed a bidirectional two-sample MR (TSMR) to elucidate directional causality and causal effects of GM on AMD progression. By leveraging the random assortment of genetic variants during meiosis, MR facilitates causal inference comparable to that of a randomised controlled trial, thereby reducing the impact of confounding factors and the bias of reverse causation that often arise in observational studies. More importantly, MR allows for the integration of multiple distinct GWAS cohorts, which reduces sampling bias and makes it feasible to investigate the GM role at various AMD stages. Together, we aim to depict a longitudinal atlas with stage-stratified GM signatures of the AMD trajectory through genetically predicted causality. Moreover, to further elucidate the potential mechanisms underlying the causal GM-AMD relationships, we incorporated potential mediatory candidates, including circulating metabolites, inflammatory proteins, and immune cell traits for mediation analysis.

## 2. Material and Methods

### 2.1 Overall survey design

**Figure 1:**
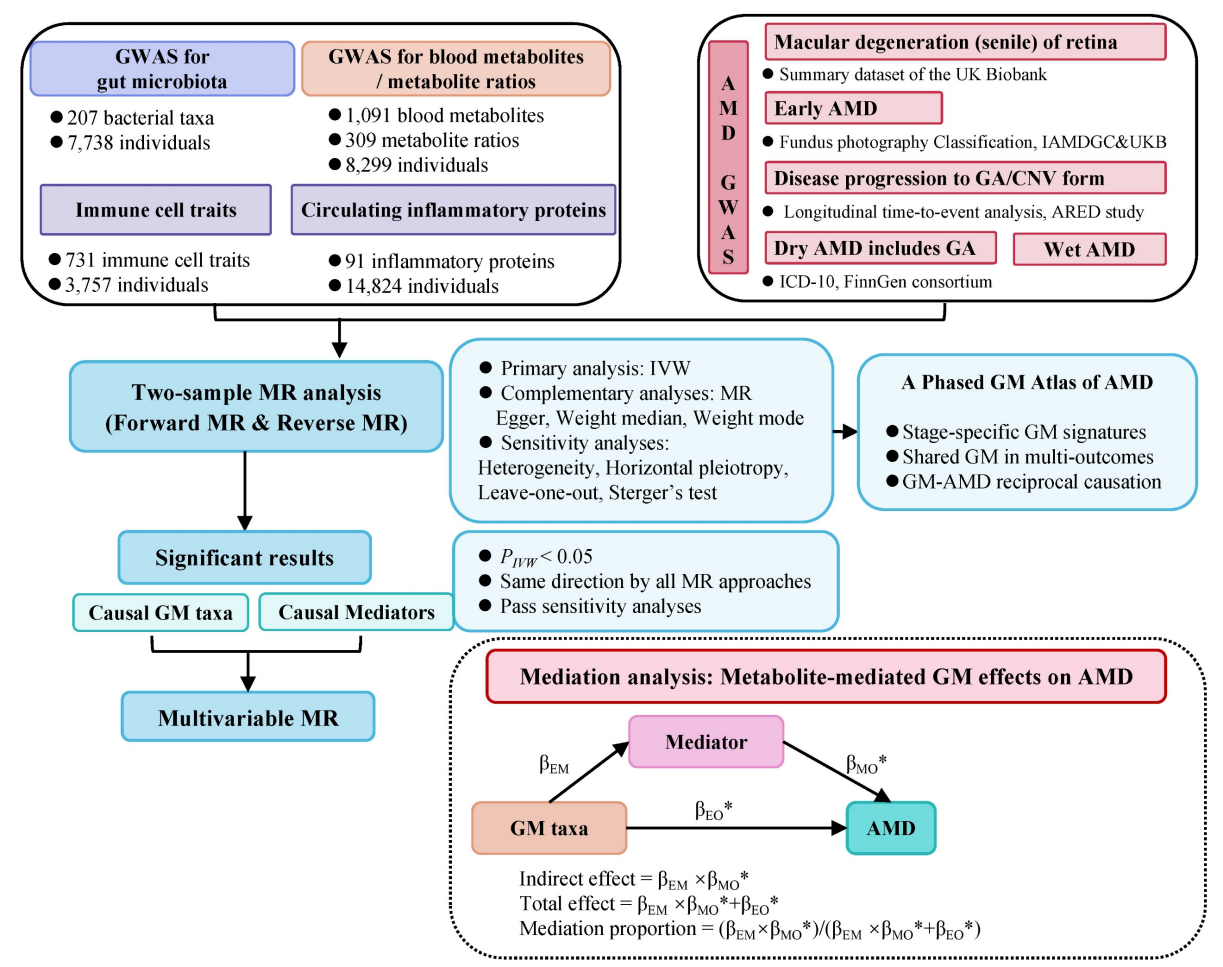
A synopsis of the research framework.

The overarching design of our study is illustrated in Figure 1. Initially, we harnessed summary GWAS data of a) exposure: the DMP cohort b) outcomes: a variety of GWAS cohorts of different AMD stages/subtypes representing the disease trajectory. We employed a two-sample Mendelian randomization (TSMR) approach, which enables investigation of causal impacts from GM exposure on various AMD cohorts (outcomes), thereby capacitating a comprehensive genetic report of the GM role in the trajectory of AMD. Subsequently, sensitivity analyses, including a reverse MR analysis, were conducted to affirm the robustness and directional consistency of the TSMR findings. To further elucidate the mediating pathways from GM to AMD, we employed a two-step mediation analysis and a multivariable MR (MVMR) approach for focused mediation analysis on specific metabolites, inflammatory proteins, and immune cell traits.

### 2.2 Data sources

#### 2.2.1 Gut microbiota

The European GWAS summary dataset for GM originates from the Dutch Microbiome Project (DMP), encompassing 7,738 individuals of European ancestry 19. The DMP examines the gut microbiome composition of participants from the Lifelines study, a renowned population-based cohort from a specific geographic region in the northern Netherlands. Utilizing shotgun metagenomic sequencing of faecal samples, the DMP incorporated a comprehensive taxonomic classification consisting of 207 GM taxa (spanning 5 phyla, 10 classes, 13 orders, 26 families, 48 genera, and 105 species).

#### 2.2.2 Various AMD stages/subtypes

Genetic variations associated with 'Macular degeneration (senile) of retina', referring to a broader definition of age-related degenerative changes in the macula lutea of the retina, were derived from a summary dataset of the UK Biobank (GCST90043776), comprising 456,348 white British participants of European descent.

Genetic variations of the onset of early AMD risk were sourced from a meta-analysis of GWAS data, including 11 data sources such as the International AMD Genomics Consortium (IAMDGC) and the UKB 20. This analysis encompassed 105,248 individuals of European ancestry, with 14,034 cases and 91,214 controls, classified based on colour fundus photography of early AMD phenotypes. Early AMD is recognized for its distinct genetic profile compared to advanced AMD at later stages. To our knowledge, it is the largest and most comprehensive dataset focused exclusively on early AMD. Detailed information of participants from these 11 population-based cohort sources, and further introduction to early AMD classification and GWAS information may be kindly found in the original publication 20.

To focus on the genetic variations representing dynamic AMD progressions and diverse AMD trajectories, we obtained genetic information from a genome-wide bivariate time-to-event analysis of 2,721 Caucasians 21, a rare GWAS dataset of a longitudinal Genome-wide bivariate time-to-event analysis on survival outcome (CNV or GA), with a mean follow-up time of 12 years in AMD patients. According to its survival outcomes, we managed to identify specific genetic variations of 'Disease progression to geographic atrophy form in AMD' (GCST005360) and 'Disease progression to choroidal neovascularization form in AMD' (GCST005358). To our knowledge, it is the first GWAS study of disease progression (bivariate survival outcome) in AMD genetic studies, providing novel insights into the genetic predisposition in AMD development 21.

Genetic variations associated with advanced AMD were sourced from the most recent data available in the Finnish Biobank (Finngen, R11), a large-scale research project that includes genome and health data from 500,000 hospital- and population-based Finnish biobank participants 22, which provided a clear delineation of the 'wet form' of AMD and the 'Dry age-related macular degeneration (includes geographic atrophy)'.

To the best of our knowledge, no prior study has enrolled sufficient patients with AMD at various stages/subtypes, nor conducted longitudinal observation tracking the entire disease progression in a large number of patients, to fulfill the investigation across the disease trajectory. Here, we integrated six GWAS datasets, each representing a distinct AMD stage or subtype. Together, utilizing a two-sample MR analysis, we may be able to conduct the investigation of GM's impacts over the disease course to some extent. On the other hand, we may reveal quite a few shared GM taxa across multiple AMD stages/subtypes with causalities in the current study. Consistent findings across multiple cohorts carry greater predictive potential and clinical relevance. Therefore, alongside revealing a stage-stratified GM signature across the AMD trajectory, this integrated MR framework may also highlight shared causal GM taxa results across multi-AMD cohorts, thereby underscoring pivotal microbial targets warranting primary attention in future observation and mechanism research in this area.

#### 2.2.3 Circulating metabolites

The GWAS summary dataset for plasma metabolites was sourced from a Canadian Longitudinal Study on Aging (CLSA) cohort, which included 8,299 individuals, encompassing 1,091 circulating metabolites and 309 metabolite ratios 23.

#### 2.2.4 91 inflammatory proteins

We integrated GWAS data on 91 circulating inflammatory proteins from a meta-analysis of 11 cohorts involving 14,824 European ancestry participants 24.

#### 2.2.5 731 immune cell traits

Summary statistical data of 731 immune-related whole-genome features were obtained from 3,757 individuals of European ancestry 25. The immune features encompass median fluorescence intensity reflecting surface antigen levels (MFI, n = 389), absolute and relative cell counts (AC, n = 118; RC, n = 192), and morphological parameters (MP, n = 32), which encompass mature stages of CDCs, monocytes, myeloid cells, TBNK (T cells, B cells, and NK cells), and Treg panels.

### 2.3 Instrumental variable selection

To identify robust instrumental variables (IVs), we applied stringent criteria. GM GWAS datasets rely on sequencing of fecal samples, which is relatively costly, leading to relatively fewer participants. Under these circumstances, the application of a relaxed p < 1×10 -5 thresholds due to the polygenic nature of GM traits and limited SNP availability at stricter thresholds (p < 5×10 -8) has been widely accepted in the majority of microbiome MR studies 26-28. Similarly, for IVs related to GM data and inflammatory proteins in the present study, we employed a relaxed GWAS significance threshold of p < 1*10^-5 for exposure-associated SNPs, ensuring adequate SNP quantity. We adhered to the conventional GWAS thresholds for SNPs associated with AMD and plasma metabolites (p < 5*10^-8). Subsequently, we employed a chained unbalanced aggregation method to filter out SNPs in linkage disequilibrium (LD) with one another, using an LD threshold of R2 < 0.001, a clumping distance of greater than 10,000 kb, and reference data from the 1000 Genomes Project European samples, thus minimizing the selection of SNPs in LD and preserving the independence of each SNP as a distinct source of genetic variation. We further selected SNPs with effect allele frequencies above 0.01 and excluded those with F-statistics below 10 to ensure the strength of the relationship between SNPs and exposure. F-statistics were derived using the formula F = Beta^2 / SE^2.

### 2.4 Statistical analyses

#### 2.4.1 Bidirectional Two-sample Mendelian Randomization

Initially, we employed a conventional two-sample univariate Mendelian randomization (UVMR) approach to assess the causal relationships between GM and AMD. For exposures with a single IV, the Wald ratio was utilized to infer causality. When exposures involved multiple IVs, we employed inverse variance-weighted (IVW), MR-Egger, Weighted median, and Weighted mode methods to ascertain causality, with IVW serving as the primary analysis. In brief, IVW synthesized SNP-specific Wald estimates using random effects to derive a consolidated estimate of causal effects. A reverse MR analysis was conducted to verify the direction of causality, specifically, to determine if an AMD subtype could potentially causally affect GM abundance. The reverse MR methods paralleled the forward MR approach, merely designating AMD as the exposure and GM as the outcome. For MR results to be considered significant, reliance on IVW analysis was paramount, and we also required that the correlation coefficients across all 4 MR methods be consistently directed to ensure the robustness of our estimations.

#### 2.4.2 Sensitivity analysis

Sensitivity analyses were conducted to ascertain the robustness of the inferred causal relationships. We assessed directional pleiotropy by examining the intercept of the MR-Egger regression; a non-zero intercept with p values < 0.05 was interpreted as a statistically significant indication of genetic pleiotropy 29. Heterogeneity among the IVs was evaluated using Cochran's Q test, where smaller p values suggest greater heterogeneity and potential for directional pleiotropy. In cases where heterogeneity was detected, a random-effects IVW analysis was conducted to yield conservative and robust estimates. In the absence of heterogeneity, a fixed-effect model was applied.

Ultimately, only those causal associations involving GM that a) exhibited no heterogeneity or pleiotropy, b) demonstrated IVW results with a significance threshold of P < 0.05, and c) showed consistent directionality of correlation coefficients across all 4 causality evaluation modes were advanced to mediation analysis.

#### 2.4.3 Mediation analysis

Mediation analysis was conducted to elucidate the potential mechanisms underlying the causal relationship between GM and AMD through circulating metabolites. We applied a two-step Mendelian randomization 30, enriched with the multivariable Mendelian randomization (MVMR) 31.

Initially, the causal relationships between GM and metabolites were assessed using two-sample MR methods to estimate the effect size β_EM_. Subsequently, the causal relationships between metabolites and AMD were evaluated to estimate the effect size β_MO_. The directions of β_EM_, β_MO_, and β_EO_ were tested following the logical framework outlined below. Lastly, MVMR was utilized to evaluate the causal effects of mediators on outcomes (β_MO_*), adjusting for exposure effects (β_EO_*) 32. Mediators with a p-value <0.05 in the MVMR-IVW were considered as the final causal mediators. The indirect effect of exposures on AMD through each mediator was calculated as the product of β_EM_ and β_MO_*, while the direct effect of exposures on AMD was β_EO_*. The total effect was determined as the sum of the direct and indirect effects (β_EM_ × β_MO_* + β_EO_*). Moreover, the proportion of mediation was calculated as the ratio of the indirect effect to the total effect ([β_EM_ × β_MO_*] / [β_EM_ × β_MO_* + β_EO_*]).

The correlation coefficients of the indirect effect, direct effect, and total effect must be consistent or adhere to the following logic: if the total effect β_EO_ is positive, then both β_EM_ and β_MO_ should be either positive or negative; if the total effect β_EO_ is negative, then β_EM_ and β_MO_ should be of opposite signs. Mediators that do not conform to this directional logic were excluded from further mediation analysis.

#### 2.4.5 Software

All analyses were conducted in the R Studio environment, utilizing R version 4.3.1. We employed the R packages “TwoSampleMR,” “MendelianRandomisation,” and “MVMR”.

## 3. Results

The GWAS source information was displayed in Table 1, and the number of SNPs used as IVs was presented in Table S1. All SNPs exhibited satisfactory validity, and all F values were greater than 10, as shown in Table S1.

### 3.1 Bidirectional MR results of causal 'GM-AMD' links

**Figure 2:**
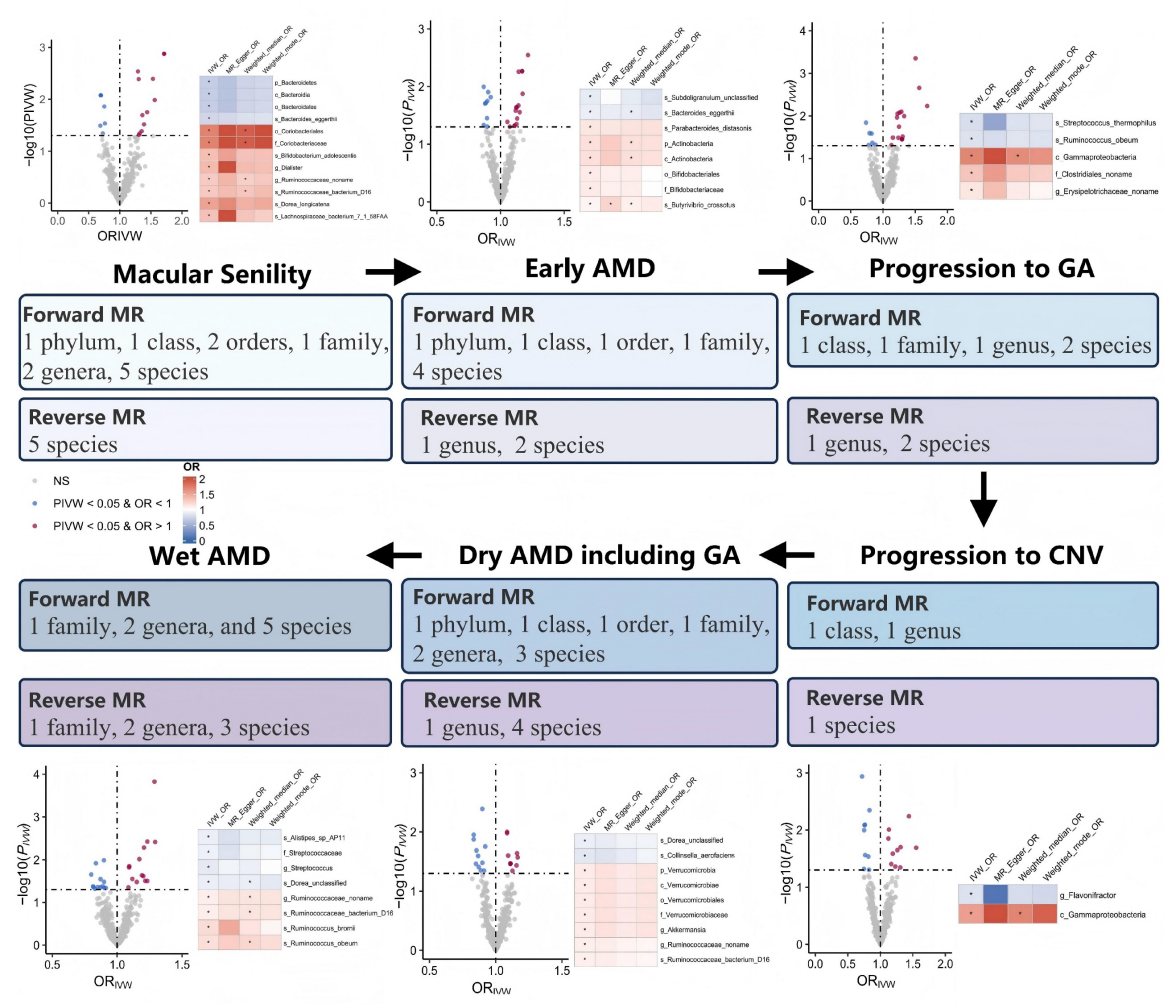
Overview of MR analyses estimating causal GM taxa and AMD across various stages. Volcano plots exhibit the causal impact of gut microbiota on AMD from the inverse variance weighted (IVW) method. The X-axis represents the logarithmic odds ratio (OR), and the Y-axis represents the -log10(PIVW). The exposure with PIVW < 0.05 and OR >1 is indicated in red, while the exposure with PIVW <0.05 and OR <1 is indicated in blue. Heatmaps outline the significant gut microbiota identified in forward MR analysis.

We identified 12/8/5/2/9/8 GM taxa associated with macular degeneration (senile) of retina, early AMD, disease progression to GA/CNV, dry AMD includes GA, and wet AMD, respectively, in the forward MR analysis (Figure 2).

**Figure 3:**
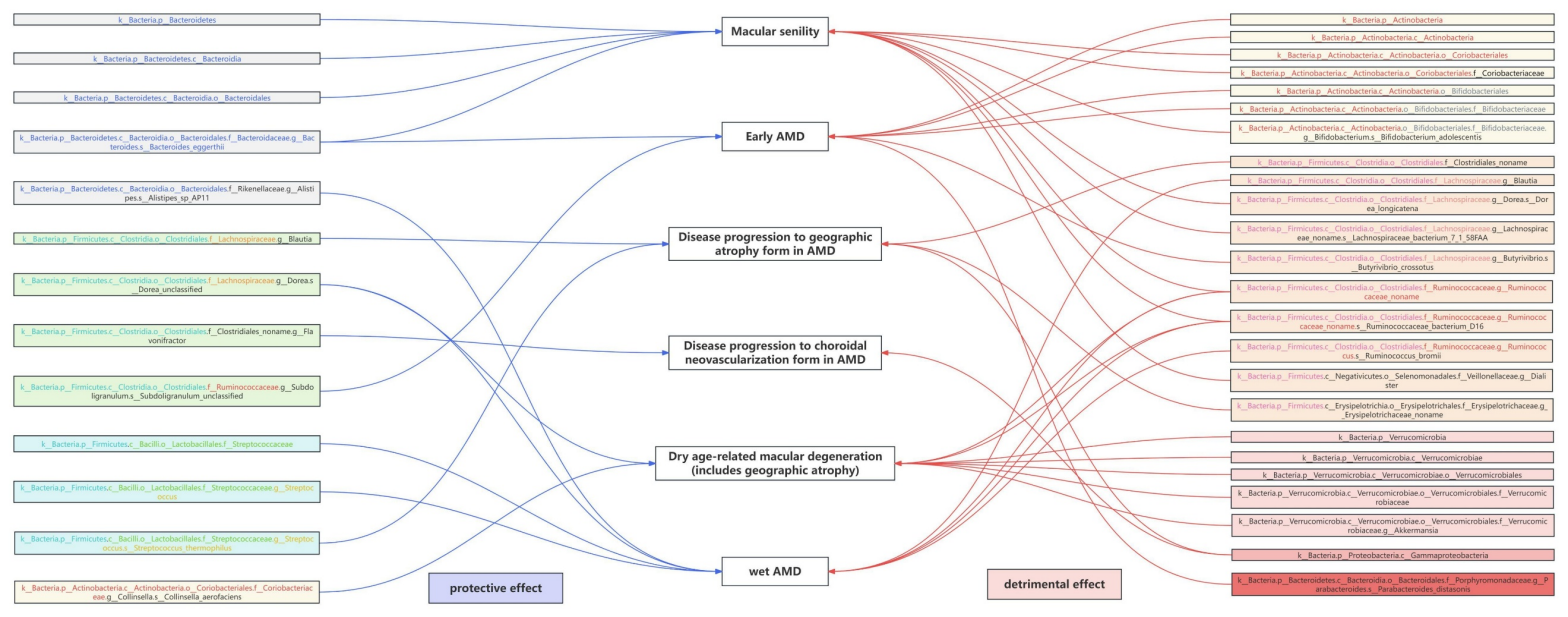
Genetically Predicted Causal beneficial or detrimental GM taxa associated with AMD at different stages.

Notably, *g.Ruminococcaceae noname* and its subordinate *s.Ruminococcaceae bacterium D16* were the only shared GM taxa of triple AMD stages with consistent and positive associations (all *P*_ivw_ values < 0.05, OR > 1, Table 2). We also revealed another 4 specific GM taxa shared from dual AMD stages. *C.Gammaproteobacteria* is one contributing taxon for both disease progression to GA and CNV (OR: 1.687, 95% CI: 1.163-2.446; OR: 1.552, 95% CI: 1.070-2.251). On the other hand, *s.Dorea unclassified* exhibited as a protective taxon for the two advanced events (wet AMD, OR: 0.898, 95% CI: 0.828-0.975; dry AMD includes GA, OR: 0.899, 95% CI: 0.837-0.967). Meanwhile, s.Bacteroides eggerthii was also revealed as a potential protective taxon for macular degeneration (senile) of retina and early AMD risk (macular degeneration (senile) of retina, OR: 0.751, 95% CI: 0.599-0.943; early AMD, OR: 0.903, 95% CI: 0.834-0.978). Additionally, *s.Ruminococcus obeum* has divergent impacts on disease progression to GA and wet AMD (disease progression to GA, OR: 0.824, 95% CI: 0.695-0.977; wet AMD, OR: 1.171, 95% CI: 1.039-1.320). A complete stage-stratified causal GM signature predicted by forward MR may be found in Table S1 & Figure 3.

When AMD served as the exposure variable, the reverse MR analysis revealed 23 significant correlations between AMD and GM, including 4 pairs of GM taxa results shared from dual AMD stages, suggesting the modification effects of AMD on microbial shifts (Table S2). Crucially, the bidirectional MR analyses revealed several instances of reciprocal causality warranting particular attention (Table 3). Notably, the abundance of the disease-contributing GM taxa, *g.Ruminococcaceae noname* and its subordinate *s.Ruminococcaceae bacterium D16* for dry/wet AMD may also be elevated by both dry and wet AMD (all OR > 1, all P*_IVW_* < 0.05 in bidirectional MR analyses). Similarly, *g.Erysipelotrichaceae_noname*'s dual role of both a mediator and amplifier was also noticed in disease progression to GA (forward MR, OR: 1.132, 95% CI: 1.001-1.280; reverse MR, OR: 1.299, 95% CI: 1.033-1.634). Interestingly, *f.Streptococcaceae* and its subordinate *g.Streptococcus* exhibited discordant bidirectional effects with wet AMD (forward MR, OR < 1; reverse MR, OR > 1, all P*_IVW_* < 0.05). Specifically, *s.Streptococcus thermophilus* (under *g.Streptococcus*) may reduce the risk of GA development (forward MR, OR < 1), while macular degeneration (senile) of retina may also decrease its abundance (reverse MR, OR < 1).

### 3.2 Bidirectional MR results of causal 'circulating metabolite-AMD' links

**Figure 4:**
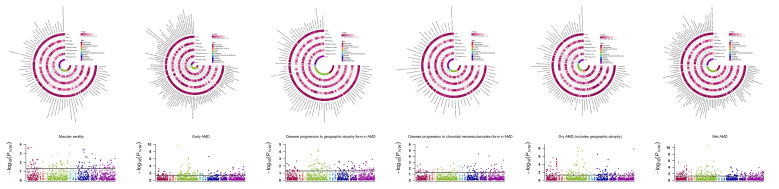
The upper part is the Circular heatmap of causal circulating metabolite levels/ratios of AMD. From the outside to the inside, they are, respectively, the name of metabolites, the *P* value and OR based on the IVW, MR Egger results, Weighted median, and Weighted mode approaches. The innermost circle exhibits 12 categories of causal metabolites including amino acids, carbohydrate, cofactors and vitamins, energy, lipid, nucleotide, partially characterized molecules, peptide, unknown metabolite, xenobiotics, and metabolite ratios; The lower part is the Manhattan plot of MR results for all 1400 metabolite levels/ratios of AMD, with the horizontal black lines indicating significant associations at *P_IVW_*<0.05.

We identified 71/104/79/54/77/106 genetically predicted causal circulating metabolites, categorized into 11 significant categories including amino acids, carbohydrates, cofactors and vitamins, energy, lipid, nucleotide, partially characterized molecules, peptides, xenobiotics and metabolites ratios, as potential mediators for macular degeneration (senile) of retina, early AMD, disease progression to GA/CNV, and dry/wet AMD, respectively (Figure 4). A complete stage-stratified causal metabolite signature may be found in Table S3, and the reverse MR results of AMD impacts on circulating metabolites were exhibited in Table S4.

### 3.3 MR results of causal 'immune cell trait/inflammatory protein-AMD' links

In the same way, we identified 31/78/22/32/46/52 genetically predicted causal immune cell traits as potential mediators for macular degeneration (senile) of retina, early AMD, disease progression to GA/CNV, and dry/wet AMD, respectively. The complete stage-stratified causal immune cell signatures are detailed in Table S5. Regarding inflammatory proteins, 3/2/3/6/12 were implicated as potential mediators for early AMD, disease progression to GA/CNV, and dry/wet AMD, respectively, while no significant mediators were found for macular degeneration (senile) of the retina. The corresponding results for inflammatory proteins are presented in Table S6.

### 3.4 Mediation analysis of causal 'GM-AMD' relationships

A two-step mediation analysis, complemented by validation through MVMR analysis, was utilized to evaluate the independent effects of candidate mediators (circulating metabolites/immune cell traits/inflammatory proteins) and exposures (GM) on AMD outcomes. This robust analytical framework ascertained a reliable prediction of causal 'GM-mediator-AMD' pathways. In accordance with the foundational principles of a two-step MR analysis for mediation analysis, we first investigated the causal relationships between causal GM taxa and causal circulating metabolites/immune cell traits/inflammatory proteins in AMD (Table S7, S8, and S9).

Then, based on significant results of the preliminary 2-sample MR analysis, the MVMR approach was employed to ascertain the independent effects of candidate mediators and exposures on the AMD outcomes (Table S10). The MVMR analysis pinpointed 17 key metabolites established as significant mediators of the causal relationships between GM and AMD. Among these, 6 metabolite levels/ratios, N-acetylputrescine, 2-o-methylascorbic acid, 1-oleoyl-2-arachidonoyl-GPE (18:1/20:4), Glycochenodeoxycholate glucuronide (1), Imidazole lactate, and Androsterone glucuronide to etiocholanolone glucuronide ratio were shared mediators in multiple 'GM-AMD' links, warranting specific attention. As for immune cell traits, CD20 on unswitched memory B cell, CD45RA on resting CD4 regulatory T cell, and Effector Memory CD8+ T cell %CD8+ T cell were identified as causal candidates mediating GM's impacts on AMD (Table S10).

## 4. Discussion

The present study is the first report of a longitudinal GM atlas with stage-stratified GM signatures of the AMD trajectory by genetic causal inference. Leveraging Mendelian Randomization analysis in multi-GWAS datasets, we managed to carry out a comprehensive investigation on the gut microbiota exposure impacts throughout the AMD course for the first time. Stage-specific GM profiles revealed phase-specific microbial contributions to various AMD stages/subtypes, whereas conserved causal GM-AMD relationships across multiple disease stages implied pan-disease GM pathophysiological mechanisms. Crucially, shared GM taxa consistently identified across multiple AMD cohorts possess heightened predictive value and clinical relevance, highlighting pivotal microbial targets for future research. Furthermore, we pinpointed specific circulating metabolites and immune cell traits that mediate the impact of GM on AMD, elucidating underlying mechanisms of the 'GM-AMD' link. Together, this cautious genetic evidence provides a global perspective of the 'GM-AMD' relationship throughout the disease course by multi-omics integration, providing testable hypotheses and theoretical evidence for microbiome-targeted AMD interventions.

### 4.1 Reciprocal causations and bidirectional synergy loops

Among the unprecedented insights from this study, the identification of GM taxa exhibiting bidirectional causal relationships with AMD may stand out as a pivotal discovery (Table 3). Notably, *g.Ruminococcaceae noname* and its subspecies *s.Ruminococcaceae bacterium D16* emerged as the sole GM taxa shared across triple AMD stages. Strikingly, we also revealed that both dry and wet AMD significantly contribute to the abundance increase of these taxa (dry AMD: β = 0.105, *P_ivw_* = 0.007; wet AMD: β = 0.078, *P_ivw_* = 0.025), while conversely, higher abundances of these taxa exacerbate AMD risks (β = 0.083-0.089, *P_ivw_* < 0.05). A similar bidirectional pattern was observed for *g.Erysipelotrichaceae noname*, which both drives and is amplified by AMD progression to GA (β = 0.262 exposure, β = 0.124 outcome; *P* < 0.05). These reciprocal pathogenic interplays underscore their dual role as mediators and amplifiers of AMD pathogenesis, which complements the traditional unidirectional "gut-to-retina" paradigm and suggests a self-perpetuating disease-microbiome feedback loop, warranting particular attention in future mechanism and intervention studies.

While bidirectional causality highlights a microbiome-disease codependency, the results of the *Streptococcaceae* family exemplify a more nuanced interplay—where taxonomic hierarchy and strain-specific functions dictate opposing roles in AMD pathogenesis. The *f.Streptococcaceae* encompasses diverse genera and species, with *g.Streptococcus*—its largest genus comprising over 100 species—exhibiting a dichotomy between pathogenic (e.g., *s.Pneumoniae*) and nonpathogenic strains (e.g., *s.Streptococcus thermophilus*, a well-characterized probiotic in the dairy industry). Forward MR identified *f.Streptococcaceae*, *g.Streptococcus*, and *s.Streptococcus thermophilus* as causal protective taxa against AMD (β = -0.091, -0.137, and -0.234, respectively; *P* < 0.05). Interestingly, reverse MR unveiled a paradox: genetic predisposition to AMD increases the abundance of *f.Streptococcaceae* (β = 0.050) and *g.Streptococcus* (β = 0.053) while suppressing the probiotic *s.Streptococcus thermophilus* (β = -0.234). We noticed the graded effect sizes in forward MR (|β|: *s.thermophilus* > *g.Streptococcus* > *f.Streptococcaceae*), which might be interpreted that probiotic species like *s.thermophilus* are the primary protective drivers within this taxon. Concurrently, according to the reverse MR, we may also propose that the AMD pathogenesis may selectively deplete beneficial strains (e.g., *s.thermophilus*) while promoting non-probiotic members of the same taxonomic hierarchy—a logical assumption with critical implications for microbiome-targeted therapies, suggesting that AMD may induce subcategory-specific microbial modulation for the first time.

### 4.2 Causal GM taxa shared in multi-AMD stages

Causal GM taxa across multi-AMD stages indicated potential pan-disease pathophysiological mechanisms in AMD progression, which deserve specific attention in future mechanism studies (Table 2). *C.Gammaproteobacteria* emerged as a key driver of both disease progressions to CNV and GA in our MR analysis. This genetic prediction aligns with our prior sequencing report, the only existing literature concerning *Proteobacteria* in AMD, suggesting a *p.Proteobacteria* enrichment in wet AMD patients 8. It is noteworthy that many common human pathogens fall under the *p.Proteobacteria* (e.g., *Escherichia*, *Pseudomonas*), characterized by their outer membrane composition rich in lipopolysaccharides (LPS) 33. Our mediation analysis further suggested that *c.Gammaproteobacteria* may disrupt homostachydrine levels and glycine-phosphate ratios. This is mechanistically consistent with a metabolomic study identifying glycine and adenosine monophosphate as central nodes in AMD metabolic networks 34. Glycine deficiency has been considered a well-characterized hallmark of AMD, and Gly is a functional amino acid against LPS-induced injury and inflammatory stress 35, 36, implying a *Gammaproteobacteria*-driven shunting of glycine into glutathione synthesis to counteract oxidative stress, thereby depleting retinal glycine pools critical for AMD development.

The *p.Bacteroidetes* and most of its subordinate taxa may prevent AMD risk from multiple stages in the present MR prediction, shedding light on future interventional studies (Table S1 & Figure 2). From the experimental aspect, the *o.Bacteroidales* has been valued in protecting against early AMD pathological features induced by dietary glycemia, such as RPE hypopigmentation and atrophy 37. Our mediation analysis further suggested that N-acetyl-putrescine may mediate the protective impacts from *Bacteroidetes* and its inferior members, which could be supported by previous research implying polyamines, like putrescine, as ubiquitous cellular components regulating the biological functions of RPE cells 38, 39.

Specifically, *s.Bacteroides eggerthii* may reduce both early AMD and macular degeneration (senile) of retina risks from our findings. RPE cells are highly enriched for the proline transporter and prefer proline as a major metabolic substrate 40-42. The neural retina also absorbs and utilizes intermediates from proline catabolism, and the metabolic disruptions of proline may lead to retinal degenerative diseases, such as its hydroxylation process 43. These experimental results lend support to our mediation result of the protective impacts of *s.Bacteroides eggerthii* on macular degeneration (senile) of retina through the proline to the trans-4-hydroxyproline ratio. Therefore, our MR prediction might provide genetic evidence underscoring the '*Bacteroidetes*-AMD' association from the perspective of the retinal metabolic ecosystem and RPE-initiated macular degeneration, echoing existing experimental conclusions.

The biological functions of the *Dorea* bacteria are strain-specific and host microenvironment-dependent 44. Therefore, the association of *Dorea* with AMD differs from existing studies 45. According to our MR analysis, we revealed that a specific *s.Dorea_unclassified* as a protective taxon for both wet AMD and dry AMD includes GA, which echoes its regulation of intestinal immune responses by inducing Treg and inhibiting the differentiation and function of Th17 cells, maintaining the integrity and stability of the intestinal mucosal barrier 44.

It is also noteworthy that the *s.Ruminococcus_obeum*, a propionate-producing bacterial species 46, may exert divergent impacts on disease progression to GA and wet AMD. Previous studies have outlined the abundance alterations of several *Ruminococcus* species in AMD conditions. *S.Ruminococcus callidus* is a shared taxon enriched in Chinese and Swiss AMD cohorts, while *s.Ruminococcus gavus* may be depleted 47. Increased abundance of *s.Ruminococcus torques* has also been reported in wet AMD patients 48. Since the present study exclusively utilized European-origin data, we highly valued compatible evidence from the existing literature, especially from cross-ethics studies implying consistent AMD-associated GM, which are more likely to be relevant to disease susceptibility or pathogenesis. Echoing the abundance alterations of multiple *Ruminococcus* species reported in existing literature of AMD observation, the present study provided genetically causal evidence the diverse biological impacts of *s.Ruminococcus_obeum* in multiple AMD subtypes.

### 4.3 *Phylum Firmicutes*, a more complex role

Nearly half of the identified causal GM taxa for the AMD trajectory are subordinating taxa under *p.Firmicutes* in the present study (Table S1). Prior studies tended to propose a detrimental role of *p.Firmicutes* in AMD, merely through individual reports suggesting specific risk-enhancing taxa such as *g.Eubacterium oxidoreducens* and *g.Ruminococcaceae UCG-011* 49, 50, or the oversimplified *Firmicutes*/*Bacteroidetes* ratio in certain disease conditions. These findings have been interpreted as evidence for a broad detrimental role of *p.Firmicutes* in AMD. However, such conclusions conflate phylum-level associations with strain-specific effects, potentially oversimplifying the complex interplay between gut microbiota composition and retinal pathology. Our comprehensive MR analysis across the full AMD disease continuum reveals a more nuanced picture: *p.Firmicutes* harbors both risk-amplifying (1 family, 3 genera, and 6 species) and protective taxa (1 family, 2 genera, and 4 species), depending on taxonomic resolution and disease stage, suggesting a hierarchical duality within *p.Firmicutes*, where divergent effects of its constituent taxa collectively shape AMD pathogenesis. This taxon-specific dichotomy complements the oversimplified 'phylum-level harm' paradigm and underscores the need for strain-resolution analyses in gut-retina axis research.

### 4.4 Understudied* p.Actinobacteria* and *p.Verrucomicrobia* in the AMD field

The *p.Actinobacteria* and *p.Verrucomicrobia* have received comparatively less attention in AMD research, with scant literature beyond a single report indicating an increased abundance of *Actinomycetaceae* and *Actinomyces* in the nasal and oral microbial communities of wet AMD patients 51. Our study now reveals their distinct pathogenic contributions: *p.Actinobacteria* and its constituent taxa exhibit robust associations with macular degeneration (senile) of retina and early AMD, while *p.Verrucomicrobia* specifically targets the dry AMD form (Figure 2). Further mediation analysis indicated their impacts on AMD through the modulation of key metabolites and immune cell traits (Table S10). These findings underscore the necessity of moving beyond the traditional focus on *p.Firmicutes* and *p.Bacteroidetes* to less-studied phyla in future AMD studies.

This integrative MR analysis provides robust genetic evidence predicting potential causal links between specific GM taxa and the AMD course, complementing current research gaps in the 'GM-AMD' area. However, MR findings should be cautiously interpreted as theoretical evidence predicting causal inference clues rather than definitive empirical conclusions. At present, empirical evidence in this field is scarce, with no large-scale, observational, or longitudinal cohort studies available to validate these predicted associations. Future research should prioritize well-powered prospective cohort studies or controlled clinical interventions targeting candidate GM taxa identified here, and ideally integrate with multi-omics profiling and functional experiments (e.g., germ-free animal models, faecal microbiota transplantation, targeted metabolic interventions, and immune profiling), which will be essential to confirm our MR causality, elucidate underlying mechanisms, and assess translational feasibility in AMD prevention and treatment.

### 4.5 Strengths and Limitations

The existing body of literature concerning GM-AMD encounters multiple methodological defects in addressing causal AMD-associated GM taxa and investigating their comprehensive impacts throughout the disease course, hindering further mechanism research. The present study utilized an integrated MR framework with intricate design, complementing these critical research gaps from a genetic perspective and surpassing the current literature in at least 5 ways: a) The Dutch Microbiome Project (DMP) provides a shotgun metagenomic sequencing of faecal samples, which enables GWAS data of GM identification to the species level. Precise identification of specific GM taxa is pivotal to future mechanism research. b) We leveraged a diverse array of GWAS datasets of AMD encompassing the disease trajectory. The two-sample MR design enables us to investigate causal impacts from GM exposure to various AMD cohorts representing different AMD stages/subtypes. Consistent results across various cohorts are particularly noteworthy, which can also enhance the genetic prediction power. c) The majority of previous studies in this field are limited to sequencing reports of GM abundance alterations in AMD conditions, where abundance change may not be directly interpreted as the biological function of GM to AMD. The MR analysis assumes the linearity in causality to predict the GM's biological impacts on AMD. d) The mediating role of circulating metabolites was also predicted through robust mediation analysis of MVMR. Causal 'GM-metabolite-AMD' pathways outlined in the present study enhance the investigation of the biological functions of GM in AMD. e) The Bidirectional MR approach raised the innovative proposal of a disease-microbiome feedback loop as a pivotal discovery for the first time. Additionally, we filtered causal results a) with no heterogeneity nor pleiotropy; b) the IVW results reached a significance threshold of P < 0.05; c) the direction of the correlation coefficients remained consistent across all 4 MR modes evaluating causality. We believe such a strict analytic framework ensures the robustness of our MR results.

Meanwhile, several limitations merit consideration. First, the GWAS data were derived almost exclusively from European ancestry. While this homogeneous design reduces the risk of population stratification bias and strengthens internal validity in MR analyses, it limits the direct generalisability of our findings to other ancestral groups. Given documented ethnic variation in AMD prevalence, genetic architecture, and GM composition, future replication in ancestrally diverse cohorts or multi-ancestry MR frameworks will be important to assess both shared and ancestry-specific causal pathways. Second, for certain GM taxa, the limited statistical power of current microbiome GWASs necessitated the use of a relaxed genome-wide significance threshold (p < 1×10⁻⁵) to ensure at least 3-5 independent SNPs for robust estimation. While this approach is supported and accepted by precedents in this field 26-28, and we followed a restricted selection of SNPs with F-statistics > 10, applied stringent LD clumping, and conducted multiple sensitivity analyses, the potential for weak instrument bias and residual horizontal pleiotropy may not be completely ignored. Third, MR findings should be cautiously interpreted as theoretical evidence-predicting causal inference clues rather than definitive empirical conclusions. Future studies integrating large-scale multi-ancestry cohorts, high-resolution metagenomics, longitudinal observational follow-up, and functional experiments will be essential to validate genetically predicted causality and enhance the translational potential of our MR findings.

## 5. Conclusions

This study marks a pioneering effort in identifying causal AMD-associated GM taxa across the entire disease trajectory, offering unprecedented insights into a longitudinal causality-robust GM-AMD association mapping and stage-specific GM signature for diverse AMD stages/subtypes. Bidirectional results complement the traditional unidirectional "gut-to-retina" paradigm and suggest a self-perpetuating AMD-microbiome feedback loop. Further mediation analyses unravelled potential mechanisms linking GM to AMD through circulating metabolites and immune cell traits, shedding light on pathways that could guide future mechanistic research.

## Supplementary Material

Supplementary tables.

## Figures and Tables

**Table 1:** GWAS data source

Items	GWAS ID	Size
Exposure		
Gut microbiota	GCST90027446-GCST90027857	7,738
Mediator		
1400 Circulating Metabolites 731 Immune Cell traits 91 Inflammatory Proteins	GCST90199621-90201020 GCST0001391-GCST0002121 GCST90274758-GCST90274848	8,299 3,757 14,824
Outcome		
Macular degeneration (senile) of retina	GCST90043776	456,348
Early age-related macular degeneration	GCST010723	105,248
Disease progression to geographic atrophy form in AMD	GCST005360	2,721
Disease progression to choroidal neovascularization form in AMD	GCST005358	
Dry age-related macular degeneration (includes geographic atrophy)	https://www.finngen.fi/en/access_resultshttps://r11.finngen.fi/	306,075
Wet age-related macular degeneration		306,042

**Table 2:** Shared GM taxa of multi-AMD stages/subtypes in forward MR analysis

GM taxa as Exposure	IVW Estimate
	OR (95%CI)
genus_Ruminococcaceae_noname	
Macular degeneration (senile) of retina	1.292 (1.091-1.529)
Dry age-related macular degeneration (includes geographic atrophy)	1.086 (1.019-1.156)
Wet AMD	1.093 (1.018-1.173)
species_Ruminococcaceae_bacterium_D16	
Macular degeneration (senile) of retina	1.304 (1.088-1.562)
Dry age-related macular degeneration (includes geographic atrophy)	1.086 (1.020-1.157)
Wet AMD	1.092 (1.017-1.173)
class_Gammaproteobacteria	
Disease progression to geographic atrophy form in AMD	1.687 (1.163-2.446)
Disease progression to choroidal neovascularization form in AMD	1.552 (1.070-2.251)
species_Bacteroides_eggerthii	
Macular degeneration (senile) of retina	0.751 (0.599-0.943)
Early AMD	0.903 (0.834-0.978)
species_Dorea_unclassified	
Dry age-related macular degeneration (includes geographic atrophy)	0.899 (0.837-0.967)
Wet AMD	0.898 (0.828-0.975)
species_Ruminococcus_obeum	
Disease progression to geographic atrophy form in AMD	0.824 (0.695-0.977)
Wet AMD	1.171 (1.039-1.320)

Footnotes: Causal associations are demonstrated by significant IVW estimates (P < 0.05), with consistent directionality of correlation coefficients across all 4 causality evaluation modes.

**Table 3:** GM taxa holding bidirectional causations with AMD

Reverse MR	AMD as Exposure	Causal GM taxa	AMD as Outcome	Forward MR	
Inverse variance weighted				Inverse variance weighted	
OR (95%CI)				OR (95%CI)	
1.107 (1.027-1.194)	Dry AMD (includes GA)	g.Ruminococcaceae noname	Macular degeneration (senile) of retina	1.292 (1.091-1.529)	

			Dry AMD (includes GA)	1.086 (1.019-1.156)	
1.081 (1.010-1.157)	Wet AMD				
			Wet AMD	1.093 (1.018-1.173)	

1.110 (1.030-1.198)	Dry AMD (includes GA)	s.Ruminococcaceae bacterium D16	Macular degeneration (senile) of retina	1.304 (1.088-1.562)	

			Dry AMD (includes GA)	1.086 (1.020-1.157)	
1.080 (1.009-1.157)	Wet AMD				
			Wet AMD	1.092 (1.017-1.173)	

1.299 (1.033-1.634)	Disease progression to GA	g.Erysipelotrichaceae noname	Disease progression to GA	1.132 (1.001-1.280)	
1.051 (1.004-1.101)	Wet AMD	f.Streptococcaceae	Wet AMD	0.913 (0.835-0.998)	
1.054 (1.005-1.106)	Wet AMD	g.Streptococcus	Wet AMD	0.872 (0.771-0.986)	
0.843 (0.718-0.989)	Macular degeneration (senile) of retina	s.Streptococcus thermophilus	Disease progression to GA	.645-0.972)	

## Data Availability

The summary statistics in this study can be found in online repositories: https://gwas.mrcieu.ac.uk/ and https://www.ebi.ac.uk/gwas/downloads/summary-statistics (last access date: 2024/9). Accession number(s) can be found in Table 1. This work originally focused on the mediatory effects of circulating metabolites solely and was requested to add alternative mediatory candidates during revision, including immune cell traits and inflammatory proteins. As such, a portion of the methodological framework and descriptive content in this article partially overlaps with related studies recently published by our research team [52, 53], as part of an integrated series of investigations.
